# Black Phosphorus Nanoflakes: An Emerging Nanomaterial for Clinical Wound Management and Biomedical Applications

**DOI:** 10.3390/ijms252312824

**Published:** 2024-11-28

**Authors:** Luke S. Smith, Hanif Haidari, Anteneh Amsalu, Gordon S. Howarth, Saffron J. Bryant, Sumeet Walia, Aaron Elbourne, Zlatko Kopecki

**Affiliations:** 1 School of Animal and Veterinary Sciences, The University of Adelaide, Roseworthy, SA 5371, Australia; smils003@mymail.unisa.edu.au (L.S.S.); gordon.howarth@adelaide.edu.au (G.S.H.); 2 Future Industries Institute, University of South Australia, Mawson Lakes, SA 5095, Australia; hanif.haidari@unisa.edu.au (H.H.); anteneh.geremew@unisa.edu.au (A.A.); 3 Gastroenterology Department, Women’s and Children’s Hospital, North Adelaide, SA 5006, Australia; 4 School of Science, College of Science, Engineering and Health, RMIT University, Melbourne, VIC 3001, Australia; saffron.bryant@rmit.edu.au (S.J.B.); aaron.elbourne@rmit.edu.au (A.E.); 5 Centre for Opto-Electronic Materials and Sensors, School of Engineering, RMIT University, Melbourne, VIC 3001, Australia; sumeet.walia@rmit.edu.au

**Keywords:** black phosphorus, 2D nanomaterials, antimicrobial, wound healing

## Abstract

Black phosphorus (BP), a two-dimensional material, has gathered significant attention over the last decade, primarily due to its unique physiochemical properties and potential role in various biomedical applications. This review provides an in-depth overview of the synthesis, nanomaterial properties, interactions, and biomedical uses of BP, with a particular focus on wound management. The structure, synthesis methods, and stability of BP are discussed, highlighting the high degree of nanomaterial biocompatibility and cytotoxicity. The antimicrobial properties of BP, including mechanisms of action and preclinical studies to date, are examined, emphasizing the effectiveness of BP against various clinical pathogens relevant to wound management. Additionally, the versatility of BP in biomedical implementations is highlighted through utilization in drug delivery, imaging, and photothermal therapy, with a focus on scalability and reproducibility with outlined future perspectives. Despite identified challenges for translation in clinical uses, BP nanomaterial has significant potential as a versatile platform in biomedical applications, especially in wound management.

## 1. Introduction

Black phosphorus (BP) is a two-dimensional (2D) material consisting of a single layer of atoms arranged in a lattice structure. The material was first discovered in 1914 and has gained significant attention for its distinctive properties for biomedical applications [1]. BP, an allotrope of phosphorus, is characterized by unique properties including high carrier mobility, strong light-matter interactions, and an adjustable band gap [2]. Unlike the other two physical forms of phosphorus, namely white phosphorus and red phosphorus, which have been extensively studied for industrial applications, BP’s two-dimensional nature gives rise to a plethora of novel electronic, optical, and mechanical characteristics, making this nanomaterial a promising candidate for various technological and biomedical applications such as photothermal cancer therapy, photovoltaics, optoelectronics, and bioimaging [3,4]. For example, BP has sparked a major interest in biomedical applications, driven by the multifunctional properties of the nanomaterial. BP has therefore emerged as a promising avenue to address the ongoing issues of antibiotic-resistant wound infection, supporting global initiatives to overcome the challenge of antimicrobial resistance (AMR).

Chronic infected wounds remain a critical research focus due to the limitations of current treatment approaches and the increasing rise of antimicrobial resistance within hospital and community settings. The growing ineffectiveness of last-resort antibiotics is further exacerbating this issue, leading to prolonged treatment regimens, extended hospital stays, increased costs, and patient suffering. As bacteria evolve mechanisms to withstand the effects of commonly used antibiotics, infections that were once easily treatable become persistent and potentially life-threatening. Recent global estimates of antimicrobial resistance burden show that it is ever increasing and has resulted in the death of over 39 million. It is estimated that antibiotic-resistant infections will kill more than 10 million people by 2050 if there are no immediate alternative solutions to treat resistant infections [5,6,7,8]. Moreover, the development of new classes of antibiotics is slow and costly, and bacteria are capable of developing resistance to new drugs over time. Therefore, innovative approaches including the development of novel antimicrobial agents, targeted therapies, and advanced drug delivery systems are essential to address this clinical problem. These alternative antibiotic-free approaches could help overcome existing resistance mechanisms, reduce reliance on traditional antibiotics, and provide more sustainable and effective treatment options.

Current research is increasingly focusing on the development, synthesis, and application of novel BP nanomaterial as a promising alternative to combat antibiotic-resistant infections [9,10,11,12,13]. BP has been recognized as an effective nanomaterial for addressing AMR through its multifaceted mechanism of action for applications in clinical wound management and drug delivery, offering a simple, safe, and efficient approach to enhancing therapeutic outcomes. To date, the preparation and applications of BP have been extensively studied and reviewed for BP applications in cancer treatment, bone degenerative conditions, and various other biomedical applications [14,15]. However, there is limited understanding of the potential role of BP in wound management. The main challenges that limit BP application in wound management include instability in the protease-rich wound microenvironment, rapid nanomaterial degradation, and inconsistent antimicrobial activity. This review focuses on the most current developments of BP for wound healing applications, limitations, potential, and prospects as a sustainable antibacterial wound care solution for clinical use.

## 2. BP Nanomaterial Structure

**Figure 1:**
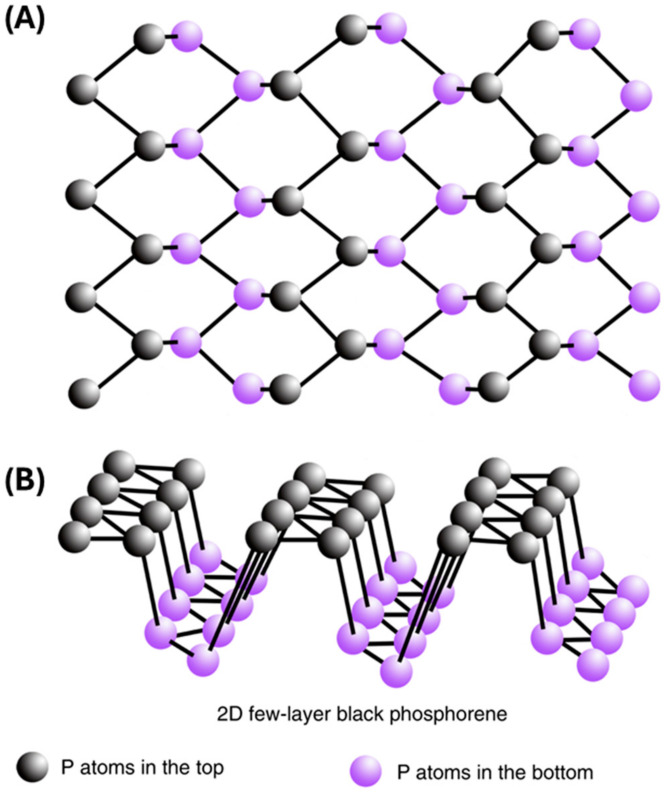
Schematic illustration of atomic structure of 2D-BP. (**A**) Top view. (**B**) 3D view. (Adapted from [29] with permission.)

The structure of BP consists of layers of phosphorus atoms arranged in a honeycomb lattice, similar to graphene. Each layer is composed of covalently bonded phosphorus atoms, forming a two-dimensional sheet. These sheets are stacked together through weak van der Waals interactions, creating a bulk crystal structure. BP is also a semiconductor with a bandgap of ≈0.3 eV for bulk and ≈2 eV for a single layer nanomaterial [16]. Moreover, the direct bandgap can be tuned by adjusting the layer number and through chemical modifications, thereby allowing different fluorescent and optical properties, which can be utilized in biomedical applications [17,18,19]. Another unique property that can be used in biomedical applications comes from BP’s ability to generate reactive oxygen species (ROS) as it degrades; the degradation and generation of ROS as a byproduct are controllable by light [20,21,22]. These unique properties also allow BP to be effectively used in biosensing, photoacoustic imaging, photodynamic therapy, photothermal therapy, and drug delivery [17,18,19]. Phosphorus, which forms BP, is a vital element in bones and constitutes approximately 1% of total human body weight [23,24]. Phosphorus is also one of the main components of nucleic acids associated with BP becoming a biocompatible material [17,25,26]. As shown in Figure 1, BP is characterized by a crystalline nonplanar structure arranged in a rigid lattice and honeycomb network connected by strong phosphorus binding and weak interlayer van der Waals forces [11,27,28,29].

BP can be readily exfoliated into ultrathin few-layer or monolayer nanosheets by breaking down the weak interlayer interactions [28]. Additionally, the thickness and distribution of BP can be further modified by changing the mechanical force, and temperature [27]. Few-layered BP has been shown to be important for both biomedical applications, including wound healing and cancer therapy, and other fields, including electronics [29]. Stacked BP nanosheets held together by weak van der Waal forces, known as few-layered BP, are highly antimicrobial to many fungi and bacteria species [30]. However, these forms of BP are unstable and degrade quickly in the air [10,20,27,30,31]. BP can efficiently be used as a photocatalyst in catalytic energy harvesting and energy conversion, hence allowing the use of BP nanomaterial in photovoltaic solar cells and energy production applications [32]. BP can be readily exfoliated into ultrathin few-layer or monolayer nanosheets by breaking down the weak interlayer interactions [30]. Additionally, the thickness and distribution of BP can be further modified by changing the mechanical force and temperature [28]. Few-layered BP has been shown to be important for biomedical applications including wound healing, anti-cancer, and electronics [31]. Stacked BP nanosheets held together by weak van der Waal forces, known as few-layered BP, are highly antimicrobial to many fungi and bacteria species [32]. However, these forms of BP are unstable and degrade quickly in the air [11,21,28,32,33]. BP can efficiently be used as a photocatalyst in catalytic energy harvesting and energy conversion, hence allowing the use of BP nanomaterial in photovoltaic solar cells and energy production applications [34].

### 2.1. Synthesis of BP Based Solutions

**Figure 2:**
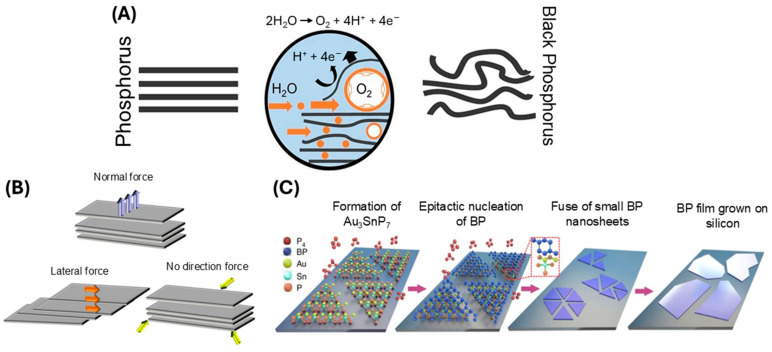
Methods of BP synthesis. (**A**) Electrochemical exfoliation (adapted from [42] with permission); and (**B**) Three types of mechanical exfoliation (adapted from [43] with permission); (**C**) Gas-phase growth with epitaxial nucleation design (adapted from [35] with permission).

Currently, there are two primary approaches of BP preparation, namely the top-down method and the bottom-up method. The top-down method uses chemical and mechanical exfoliation to break bulk BP down into nanometric sizes and includes mechanical stripping, liquid stripping, ultrasonic processing, solvothermal treatment, and blender shearing [13,31,35]. An example of the top-down method is liquid exfoliation. Liquid exfoliation utilizes solvents including dimethylformamide (DMF), N-cyclohexyl-2-pyrrolidone (CHP), N-methyl-2-pyrrolidone (NMP), dimethyl sulfoxide (DMSO), and isopropyl alcohol (IPA) [17]. One example of liquid exfoliation includes bulk BP nanomaterial being mixed with sodium hydroxide (NaOH) solvent, followed by stripping using sonication, centrifugation, washing, and redispersion in water. Compared to other reported methods, liquid exfoliation is more efficient and controllable for yielding maximum BP concentration. This method can also control the size and thickness of the BP nanosheets and is easy and simple to operate, and the BP can readily be used or stored in a solvent [36,37]. For example, Brent and colleagues used N-methyl-2-pyrrolidone (NMP) to prepare few-layered BP nanosheets via liquid exfoliation [38]. As shown in Figure 2A, the NMP and NaOH solvent, combined with ultrasonic exfoliation, results in BP formulation. Currently, studies are being undertaken to avoid the use of harsh solvents and instead use physiological solutions, which would avoid the potential toxic side effects of solvents during biomedical applications. Figure 2B shows two methods of mechanical exfoliation, combined with the method of top-down BP formulation.

The bottom-up method uses a direct chemical synthesis of nanomaterials using a particular precursor involving techniques including pulsed layer deposition and chemical vapor deposition [31]. These methods offer tunability in terms of nanomaterial size, shape, and surface properties, enabling the fabrication of BP materials tailored for specific biomedical applications [28]. Compared to liquid exfoliation, these methods are generally more costly and complicated, limiting commercial applications. One example of a bottom-up approach is gas-phase growth, shown in Figure 2C. BP synthesis is described more comprehensively in several recent reviews [35,39,40,41].

### 2.2. Stability of BP Nanomaterial

**Figure 3:**
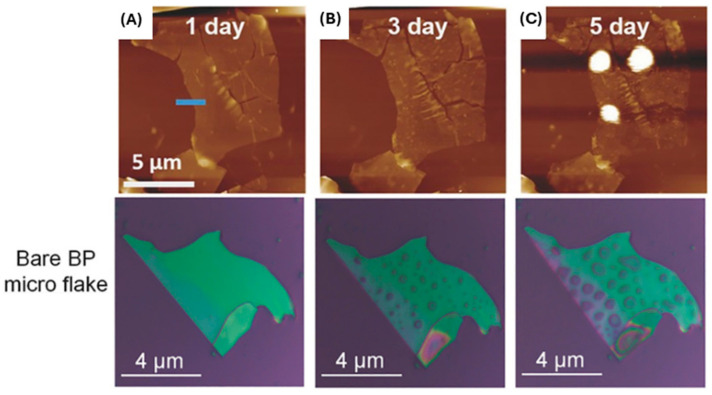
AFM images of an unmodified BP sheet exposed to air for (**A**) 1 day, (**B**) 3 days, and (**C**) 5 days; photographs of a bare BP flake exposed to air for 0, 4, and 8 days (adapted from [35] with permission).

To realize the possible applications of BP nanomaterial in a clinical setting, it is vitally important to develop facile and highly stabilized BP nanomaterial to allow for transformative impact in wound management. To date, a considerable amount of effort has been made to improve the fabrication methods of BP nanomaterial and enhance inherent BP stability. Currently, the inherent instability in ambient conditions contributes to limited progress for widespread applications. The phosphorus atom in BP has a lone pair electron that is readily disposed to oxygen molecules; this results in uncomplicated oxidation and degradation of the nanomaterial in both air and water [44]. Additionally, the high degree of BP instability is attributed to high surface-to-area ratio, valence bonds with angles of 102°, lone electron pairs, and high chemical reactivity [45,46,47,48]. This instability makes the long-term storage of BP a challenge. By itself, BP stored in ambient conditions will degrade within days; however, if layer thickness is increased, it can be stored for 2 weeks with minimal degradation [49]. A recent study has explored and illustrated the use of coatings and solvents as solutions to increase BP stability in ambient environments and therefore increase storage time significantly [50]. These coatings and solvents can allow BP to be stored for long periods of time (>3 months) [46,51]. The degradation of BP is initially slow but increases up until a saturation point, following an S-shaped growth curve (sigmoid growth curve) [46,47]. The rate of BP degradation is affected by oxygen concentration, light intensity, presence of water, temperature, and BP nanoparticle thickness [46,51,52]. As BP degrades, it forms oxidized phosphorus species [PO_x_^3−^ (x = 2, 3, 4)]. The specific species (x) is dependent on the oxidant concentration, which is advantageous, as phosphorus is essential for tissue regeneration and wound healing, and PO_x_^3−^ is highly biocompatible [9,48,53]. The effects of BP degradation can be observed using atomic force microscopy (AFM) and optical microscopy. Liquid droplets form on the surface of BP nanoflakes after less than 24 h of exposure to air, as can be observed in Figure 3 using AFM and optical microscopy. These droplets are attributed to water being adsorbed onto the surface, condensed from the ambient environment moisture [27,35,48].

The precise method of BP nanomaterial degradation has been a topic of discussion, as the method was not well understood; however, recent research has clarified this process. Castellanos-Gomez et al. (2014) suggested that the hydrophilic nature of BP nanomaterial leads to degradation [27]. These authors proposed that this hydrophilicity caused water molecules to adhere to BP nanomaterial, resulting in greater adsorption and binding energies that degrade the thin BP nanoflakes [27]. However, few-layer BP nanoflakes remained stable for several days [27]. Additionally, Wood et al. (2014) showed that after one day in ambient conditions, significant degradation bubbles formed, which increased the opportunity to form hydrophilic dipoles [48], while Favron et al. (2015) attributed BP degradation to photoinduction, which requires simultaneous interaction of light, water, and oxygen [52]. In agreement with these studies, Wang et al. (2016) demonstrated that BP degrades through contact with oxygen and only reacts with water after it has been oxidized [54]. Recent studies clearly show that the mechanism of degradation is photo oxidation-induced damages, which are accelerated in the presence of humidity [20,21,22]. Therefore, most studies to date examining BP nanomaterial degradation conclude that poor environmental stability of the BP nanomaterial limits some of the applications and clinical translation. However, the capacity to control the rate of BP nanomaterial degradation could be an advantage for certain applications. For example, for BP applications in electronics for imaging, reduced nanomaterial degradation is optimal, but for uses in biomedical applications of wound management or antimicrobials, a more rapid BP nanomaterial degradation may lead to more favorable effects on wound infection management and promotion of tissue repair/regeneration.

### 2.3. Strategies for BP Stabilization

Studies to date agree that oxidation of BP nanomaterial surface is the primary cause of degradation and that other external factors including light, water, heat, pH, and oxygen all impact the rate of BP nanomaterial degradation. Additionally, large surface area, lone-pair electrons, defects, and nanomaterial thickness all contribute to the rate of BP degradation. To date, several protective strategies have been reported to enhance the stability of BP, including surface passivation. Various techniques have been proposed and tested for effective passivation of BP via surface functionalization or coatings, including organic covalent functionalization and inorganic and hybrid organic–inorganic coatings. The coating can serve as a physical barrier preventing oxygen from reaching the surface. The coating can also bind to BP’s lone pair of electrons, preventing the oxidation reaction. For example, Illarionov and colleagues have shown that passivation layers of Al_2_O_3_ on BP considerably improve the material stability and maintain the reproducible characteristics for at least 17 months, thereby preventing reaction with ambient oxidants [55]. The use of SiO_2_ has also been widely reported [56]. In addition, covalent functionalization is a promising strategy to enhance the ambient stability of BP by forming a protective layer on the BP surface and occupying the reactive lone pairs, thereby preventing reaction with ambient oxygen and water. Chemical modifications, such as covalent bonding with functional groups or grafting with stabilizing agents, have shown success in preventing BP oxidation. Functional groups like amines or thiols can be introduced to enhance BP’s stability by inhibiting surface reactions that lead to degradation. The chemical functionalization has been greatly discussed and reviewed recently [57].

Other methods, including encapsulation with a variety of polymers, have also gained attention. For example, coating with polydopamine (PDA) can act as protective shells, providing a physical barrier that reduces BP’s exposure to degrading agents and improves the biomedical applications [58]. Researchers have also focused on incorporating BP within layered nanocomposites, including hybrid aerogels based on graphene oxide, resulting in enhanced properties and protection, offering potential for various applications [59]. These layered structures help protect BP from oxidation while preserving its electronic and photothermal properties. Layered composites are particularly useful in applications requiring sustained and controlled drug release. However, further research is required to fully understand the exact mechanisms underlying rapid degradation while modifying the structure to generate safe and sustainable BP material. Therefore, it is important to prevent the degradation of BP to enable its maximum potential for future biomedical applications. The protection and stability of the BP could significantly impact the downstream biological activity and safety.

## 3. BP Nanomaterial Biocompatibility and Cytotoxicity to Mammalian Cells and Tissues

Biocompatibility of the BP nanomaterial is an essential criterion for clinical translation and biomedical applications. Depending on the nanomaterial properties or application, BP nanomaterial has generally been shown to be a safe inorganic material with good clearance and degradation from the body without any side effects. Recent studies have shown that the short-term application of BP nanomaterial has excellent biocompatibility with a variety of cell types, including skin fibroblasts, endothelial cells, and immune cells; however, there are still concerns regarding the potential BP nanomaterial cytotoxicity, especially with prolonged exposure or at high concentrations [11,50,60]. Several factors, including the BP nanomaterial size, surface chemistry, and dispersibility, can influence BP nanomaterial interactions with biological systems, resulting in potential cytotoxic effects. Furthermore, rapid oxidation and degradation of the BP nanomaterial may lead to excessive production of reactive oxygen species (ROS), creating an oxidative environment that disrupts mammalian cell activity. There is an increased correlation between BP size and increased oxidative stress. Therefore, comprehensive cytotoxicity evaluation studies, including cell viability assays, cell proliferation assays, and assessment of inflammatory responses, in addition to safety studies in large animal models are necessary to validate the safety of BP-based biomedical devices and therapies before translation to clinical applications [11].

**Figure 4:**
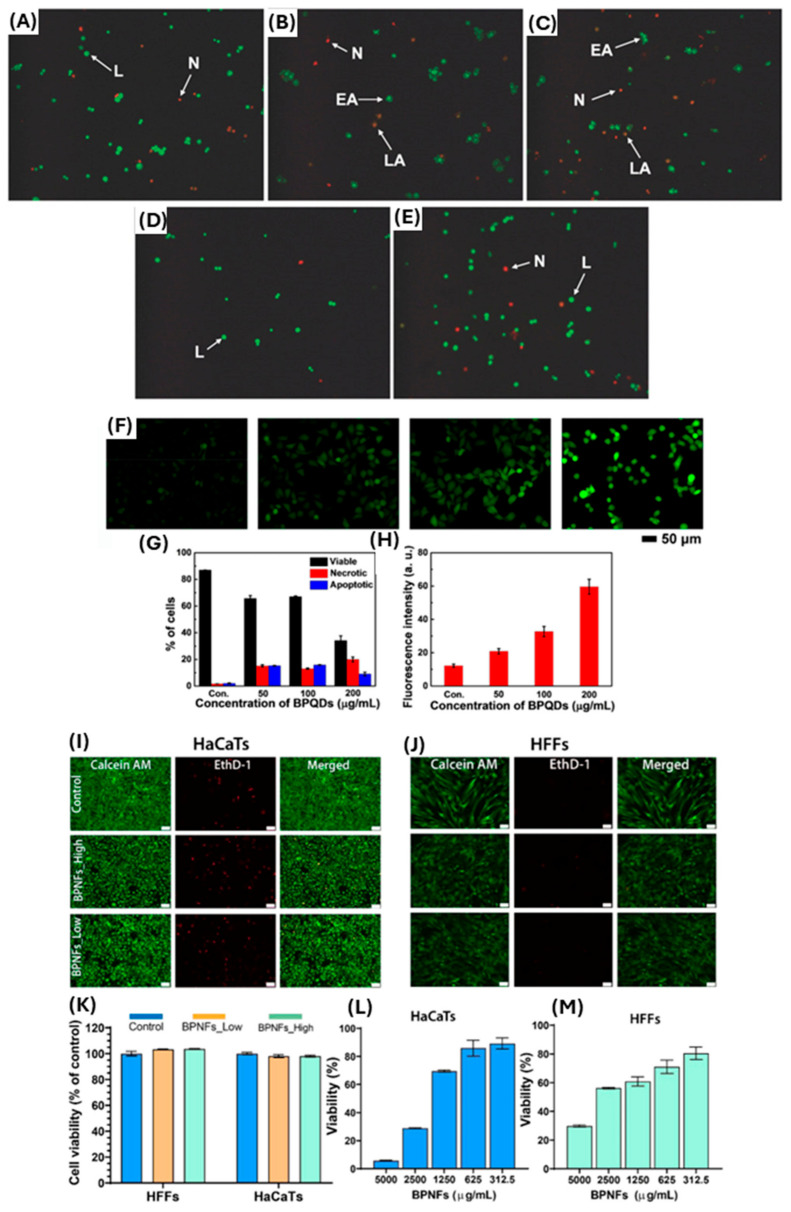
(**A**–**E**) AO/EB staining of HCoEpiC cells after 24 h exposure of layered BP observed by inverted fluorescence microscopy at various concentrations. The figure panels: (**A**) control and (**B**–**E**) 10 µg mL^−1^–50 µg mL^−1^). The arrows on these panels are as follows: L: live cells; EA: early apoptotic cells; LA: late apoptotic cells; N: necrotic cells. Adapted with permission from [63]. (**F**–**H**) show cell apoptosis and oxidative stress on HeLa cells; (**F**) intracellular ROS production in cells treated with the different concentrations (0, 50, 100, and 200 μg/mL) of BPQDs. (**G**,**H**) The corresponding analysis on percentage of cells and fluorescence intensity of ROS level, adapted from [64] with permission. (**I**–**M**) The panel shows the effect of BPNFs on in vitro skin cell viability at moderate concentrations. (**I**,**J**) Shows representative images of HaCaTs and HFFs stained with Calcein AM (green), and dead cells stained with EthD-1 (red) following 24 h of treatment with BPNFs low (1000 µg mL^−1^) and BPNFs high (1500 µg mL^−1^). (**K**–**M**) The corresponding cell viability after treatment for both skin cells at low and high concentrations compared to control. Data adapted with permission from [12]. Scale bar 50 µm. Data are shown as means ± SEM.

The results of cytotoxicity studies to date vary depending on the implementation of BP nanomaterial; therefore, previous studies can only act as a guide and provide some insight into potential cytotoxicity towards mammalian cells. The toxicological effects of various 2D materials, including BP nanomaterial, depend on their specific properties, size, and concentration [61]. Latiff et al. 2015 found that BP nanomaterial toxicity was intermediate between transition-metal dichalcogenides (TMDs) and graphene oxides (GOs). The average cell viabilities of human carcinoma epithelial cells (A549) treated with GOs and TMDs were found to be 69% and 66.75% and 93% and 85.67%, respectively [62]. Cell viability for BP treatment was measured at 81% and 68% [62]. The cell viability was tested using water-soluble tetrazolium salt (WST-1) and methylthiazolyldiphenyltetrazolium bromide (MTT) assays at 25 µg mL^−1^ exposure [62]. Studies using human epithelial colonic cells (HCoEpiC) have shown that exfoliated layered BP nanomaterial with larger lateral size and thickness has a high cytotoxicity, whereas a smaller layered BP nanomaterial showed only moderate cytotoxicity [63], illustrating the importance of BP nanomaterial size and structure for cytotoxicity effects (Figure 4A–E). In addition, Mu et al. 2017 conducted both in vitro and in vivo preclinical studies using the application of BP quantum dots (BPQD) at a concentration of 200 µg/mL and reported significantly increased apoptotic effects on HeLa cells (Figure 4F–H). Additionally, authors showed in vivo, that oxidative stress, including lipid peroxidation, reduction of catalase activity, DNA breaks, and bone marrow nucleated cell damage, could be induced by BPQDs transiently, although these changes recovered gradually to normal levels. Authors concluded that no apparent pathological damage was observed in any organ, thereby showing no long-term toxicological response due to BPQD application [64]. More recently, a study by Virgo et al. 2023 demonstrated that concentrations of BP nanoflakes at <1250 µg mL^−1^ had a >70% metabolic cell viability in human skin keratinocytes and fibroblasts, whilst higher concentrations of BP nanomaterial resulted in greater mammalian cell death in vitro (Figure 4I–M) [12]. This study also showed that topical application of BP nanoflakes at 1250 µg mL^−1^ resulted in reduced *Staphylococcus aureus* wound infection and subsequent improved wound repair, with no other adverse effects noted on animal wellbeing, hence suggesting potential safe application of BP nanoflakes for wound management. Consequently, to date, there are numerous studies affirming BP nanomaterial as a non-toxic material fora range of human cells at certain concentrations, while other studies still suggest potential toxicity and cell death. A key conclusion in preclinical studies to date is that BP nanomaterial toxicity is highly dependent on size and concentration, as well as whether the material is applied in conjunction with other products. Together this highlights the need for further research, as the specifics of BP nanomaterial application can be a determining factor of toxicity towards mammalian cells.

Additionally, long-term studies on relevant animal models are required to accurately assess the biocompatibility, biodistribution, and biodegradation of BP nanomaterial and the potential for eliciting immune responses or tissue damage that may be favorable (e.g., during wound infection) or limiting (promoting inflammation and delaying tissue regeneration). Currently, there are limited in vivo studies assessing the biocompatibility, organ accumulation, and clearance of BP nanomaterials. One such study is Virgo et al. 2023, which showed that topical application daily to a wound for 6 days resulted in no observed toxicity to the major organs of mice (heart, kidney, liver, lung, spleen) [12]. Similar to cytotoxicity, the biocompatibility of BP nanomaterial is also dependent on size and concentration [61]. As mentioned previously, BP nanomaterial degrades to form oxidized phosphorus species [PO_x_^3−^ (x = 2, 3, 4)]. This degradation pathway is advantageous, as the phosphorus species can be utilized in essential functions and structures and is readily biodegradable, preventing long-term accumulation and potential toxicity as is often observed with other 2D materials or metallic nanomaterials, including silver, during applications in wound management [9,14,48,53].

While BP nanomaterial exhibits significant potential for biomedical applications, its toxicity must be comprehensively investigated to develop a biocompatible nanomaterial for wound healing applications. Systematic research on BP nanomaterial toxicity is currently limited to in vitro studies, examples of which are summarized in Table 1. As shown in Table 1, depending on the types of BP and the test performed, there is a broad range of concentrations that are shown to be tested against different cell lines. Understanding the mechanisms underlying BP nanomaterial cytotoxicity is crucial for mitigating risks and ensuring safe usage and clinical translation. Strategies used in nanomedicine including material surface modification, dose optimization, and the development of biocompatible formulations can help harness the benefits while minimizing any adverse effects to the host. Accordingly, there is an emphasis on applications of BP nanomaterial in various drug delivery systems, which can facilitate slower degradation, increased stability, and sustained effects. This approach helps mitigate potential BP nanomaterial toxicity through controlled release, reducing direct exposure to mammalian cells and enhancing material stability. Encapsulating BP nanomaterial in biocompatible carriers or coating it with protective layers are some of the strategies that could be employed to prevent rapid BP nanomaterial degradation and excessive reactive ROS production [65,66]. These approaches not only minimize BP nanomaterial cytotoxicity, but also ensure targeted delivery to specific tissues or cells, thereby reducing the overall dosage required and limiting potential side effects.

## 4. Potential Biomedical Application of BP Nanomaterial

BP nanomaterial demonstrates an exceptional potential in biomedical applications due to the unique properties that offer promising clinical advantages. In drug delivery, BP nanomaterial can serve as a platform for controlled-release systems that improve therapeutic efficacy and safety by allowing precise release kinetics tailored to treatment needs. Photothermal and photodynamic properties of BP material further extend potential clinical applications to imaging and phototherapy, enabling precise bacterial targeting, tumor ablation, and real-time monitoring of therapeutic effects. BP’s inherent antimicrobial activity also positions this material as a valuable tool in the fight against drug-resistant bacterial infections. Unlike traditional antibiotics, BP does not rely on specific bacterial metabolic pathways, which reduces the likelihood of resistance development—a growing limitation with conventional antibiotics.

Additionally, BP nanomaterials demonstrate high biocompatibility, promoting cell proliferation and migration, which makes BP-based materials highly suitable for clinical applications during complex wound management. BP-based dressings can accelerate tissue regeneration and recovery, potentially reducing healing times for chronic wounds. Compared to traditional materials, BP’s ability to integrate multiple therapeutic functions—including drug delivery, antibacterial action, and wound healing—in a single platform highlights the advantage of BP over traditional materials, which typically fulfill only one role at a time. BP’s multifunctional capabilities and versatility therefore support the use of this material as a next-generation material in biomedical applications, particularly for tackling complex medical challenges like chronic infections and drug-resistant pathogens.

### 4.1. BP Nanomaterial and Drug Delivery

BP nanomaterial offers a multifaceted approach to enhance therapeutic outcomes. Its layered structure allows for high drug loading capacity, while material responsiveness to environmental stimuli including pH, light, and temperature can be leveraged for precise, targeted, and on-demand drug release. By encapsulating drugs within BP nanosheets, or incorporation into BP-based nanomaterial carriers, it is possible to achieve sustained and targeted drug delivery, reducing the frequency of administration and minimizing systemic side effects. Moreover, the degradation rate of BP nanomaterial can be finely tuned to match therapeutic needs, ensuring that drugs are released at the optimal rate and concentration. Therefore, protective coatings or encapsulation within biocompatible materials can further enhance the stability of BP nanomaterial, preventing premature degradation and controlling release kinetics.

The large surface area of BP nanosheets has been shown to provide an attractive strategy for efficient and high drug loading and controlled release [31,71,72]. This characteristic is particularly critical in targeted drug delivery systems, where the precise delivery of therapeutic agents to specific tissues or cells is essential for minimizing side effects and optimizing efficacy [31,72], as summarized in Table 2. BP nanomaterial can be used for delivery of small or large drugs, nanoparticles, and other macromolecules that are effective for a variety of diseases, predominantly for cancer and wound applications. Moreover, the tuneable bandgap of BP nanomaterial allows for fine-tuning of the release kinetics of drugs, ensuring a sustained and controlled delivery over time [31,71,73]. This capability addresses challenges associated with conventional drug delivery systems, where rapid drug release can lead to suboptimal therapeutic outcomes and potential side effects [73].

The controlled release from BP nanomaterial platforms can be tailored to match the desired therapeutic window, enhancing the overall effectiveness of drug treatments. Functionalization of BP nanomaterial with biocompatible polymers or targeting ligands has been shown to further enhance its drug delivery capabilities [13,31,74,75,76]. This functionalization not only improves the biocompatibility of BP nanomaterial but also facilitates specific targeting of drugs to diseased tissues or cells. By modifying the surface of BP with ligands that recognize specific receptors on target cells, drug-loaded BP nanoparticles can selectively accumulate at the site of interest, improving drug delivery precision and minimizing systemic exposure [31,45].

**Table 2:** Examples of studies where BP has been used to deliver other materials/drugs and the area of application.

Treatment Delivered	Application	Delivery Method	Reference
Doxorubicin	Posterior capsule opacification, Cancer treatment	Implantation, or intratumuoral injection, followed by NIR	[67,76]
Epigallocatechin gallate	Diabetes with infected burn wounds healing	Hydrogel applied topically, followed by NIR	[77]
Polycaprolactone	Bacterial infection with fracture healing	Implantation, followed by NIR	[78]
Indocyanine green	Periodontal disorders treatment	Injection, followed by NIR	[79]
Zinc oxide	Implant-associated infections treatment	Titanium surface coating, implantation, followed by NIR	[58]
Gold	Pathogen bacteria treatment	Topical, followed by NIR	[80]
Silver	Resistant bacterial infection treatment	Subcutaneous injection, followed by NIR	[10]
Hydroxyapatite	Biofilm ablation and bone fracture healing	Implantation, followed by NIR	[81]
Silk fibroin	Skin infection wound healing	Topical application of dressing (Sponge), followed by NIR	[70]
Polyetheretherketone/Polytetrafluoroethylene	Artificial bone joint material infection prevention	Implantation	[82]
Mitoxantrone hydrochloride and hyaluronic acid	Cancer therapy	Intravenous injection, followed by NIR	[71]
Oxaliplatin (1,2-diaminocyclohexane) platinum (II) (DACHPt)	Cancer Therapy	Cultured/incubated with tumour cells, followed by NIR	[72]

A recent study by Fu et al. used ε-poly-l-lysine (ε-PL)-engineered BP nanosheets with dual delivery systems to target bacterial membranes for on-demand application [69]. The study integrated antimicrobial peptides alongside photothermal agents to construct BP nanomaterial with synergistic properties and high drug loading capacity. The study reported that after application to the infected site, the smart dual drug-delivery complex could closely interact with bacterial surfaces based on charge difference, leading to rapid membrane disintegration. Subsequently, in situ hyperthermia generated by BP nanomaterial can further support the complete eradication of pathogenic bacteria. In addition, the BP has also been doped with silver nanoparticles (AgNPs) to synergistically enhance the antibacterial effect, serving as a delivery mechanism for the controlled release of silver [83]. This follows an identical approach as several studies have demonstrated excellent antimicrobial activity of silver nanoparticle against a broad range of pathogenic bacteria [84,85]. Silver nanoparticles (AgNPs) remain prominent nanomaterials utilized for their antimicrobial properties in medical applications, particularly in wound management. AgNPs have been extensively studied and employed due to their broad-spectrum antibacterial activity, which arises from multiple mechanisms [86]. However, it is well documented that AgNPs and other antimicrobial agents (e.g., graphene oxide) act non-specifically and are susceptible to damaging both bacterial and healthy mammalian cells. For example, silver ions do not target infected areas exclusively, which can sometimes lead to cytotoxicity and tissue irritation in sensitive applications. This remains the biggest drawback of AgNP materials for wound management. In comparison, BP offers more opportunities for broader biomedical applications due to the photothermal activity, which can be controlled and localized by NIR light [65]. This enables BP to target specific areas of infection without affecting surrounding healthy tissue, as demonstrated by clearing and promoting MRSA-infected wounds in diabetic rats [65]. In addition, BP has many other properties desirable for wound care applications. For example, the desirable photoresponsive properties of BP nanomaterial have also opened an exciting photo-responsive drug delivery system for various conditions. The near-infrared (NIR) absorbance of BP nanomaterial adds another layer of sophistication to the design of novel drug delivery systems. Photoresponsive drug delivery, where drug release is triggered by NIR light, enables control of drug release [31,71,73,75,76]. This feature is particularly advantageous in scenarios where precise timing and location of drug delivery are critical. For example, in cancer therapy, photoresponsive drug delivery systems that incorporate BP nanomaterial can be designed to release therapeutic agents specifically within tumor tissues upon exposure to NIR light, minimizing damage to healthy surrounding tissues [31,45,71,73].

### 4.2. BP Nanomaterial for Biosensing and Imaging Applications

BP nanomaterial has been employed as a biosensing platform for sensitive diagnosis of cancers and other conditions including infection. Additionally, BP nanosheets have been used for electrical, electrochemical, fluorescent, and colorimetric biosensing due to their excellent electrical and electrochemical properties [87]. The excellent intrinsic properties of the BP nanomaterial position this versatile material for various imaging purposes, each contributing to rapid and reliable detection for improving early diagnosis. The optical properties of BP nanomaterial are dependent on the extent of BP nanosheet deformation, and the optical bandgap can be tuned from 0.38 to 2.07 eV [31,88]. Additionally, BP nanomaterial optical response can be regulated by controlling the degree of deformations. Studies to date have shown that the number of stacked layers not only affects the ability of electrons to interact, but also controls the excitonic effects and optical spectra of BP nanomaterial [31].

**Figure 5:**
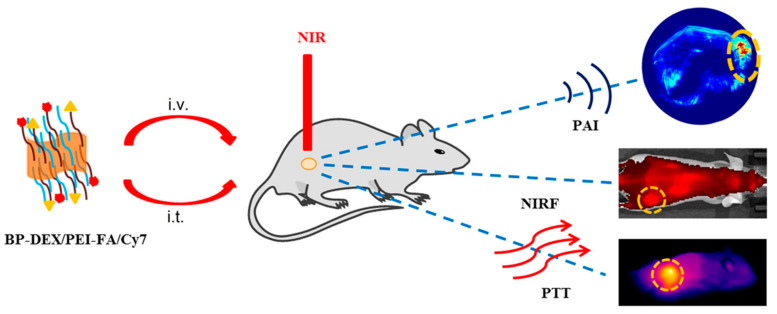
BP (BP-DEX/PEI-FA/Cy7) nanoparticles and their applications in photoacoustic (PA) imaging, near-infrared fluorescence (NIRF) imaging, and photothermal therapy (PTT) of cancer (i.t.: intratumoral injection, i.v.: intravenous injection). (Adapted from [68] with permission.)

Targeted biomarkers typically exist in very low concentrations in clinical blood samples, necessitating the creation of highly sensitive and specific medical biosensors. BP nanomaterial has therefore been used as a biosensing platform for the accurate diagnosis of diseases such as cardiovascular disease and breast cancer [17]. In photoacoustic imaging, BP nanomaterial-based contrast agents leverage the high photothermal conversion efficiency of BP nanomaterial to generate detailed images with high resolution, as seen in Figure 5 [31,68].

When exposed to pulsed laser light, BP nanomaterial absorbs the energy and induces a rapid thermoelastic expansion, generating acoustic waves that can be detected and converted into detailed images [26,31,45,73]. This technique allows for deep tissue imaging, overcoming the limitations of traditional imaging methods, and holds promise in visualizing structures with high precision. Furthermore, fluorescence imaging using BP nanomaterial-based contrast agents exploits the NIR absorption and emission characteristics of BP [25,89].

Traditional fluorophores often face limitations in tissue penetration and background interference; in contrast, BP nanomaterial NIR properties enable deeper tissue penetration and reduced interference, enhancing the imaging depth and sensitivity [25,89]. This is particularly advantageous in applications where visualization of specific biological structures or processes deep within tissues is critical. Lastly, the compatibility of BP nanomaterial with magnetic resonance imaging (MRI) expands the degree of the nanomaterial biomedical applications in multimodal imaging [90,91,92]. By combining the benefits of BP nanomaterial optical and imaging properties, in combination with other MRI-guiding compounds, comprehensive and detailed anatomical information can be obtained along with functional insights [90,91,92]. This multimodal imaging approach offers a holistic understanding of biological processes, aiding in the diagnosis and monitoring of various medical conditions.

### 4.3. Photothermal Applications of BP Nanomaterial

Photothermal agents are rapidly attracting attention for a variety of drug delivery and sensing applications. The ideal photothermal agent should not only have a considerable extinction coefficient and photothermal conversion efficacy in the NIR region but also satisfy the safety aspects. BP nanomaterial has excellent NIR photothermal performance and biocompatibility. Over the years, BP nanomaterials with different numbers of layers and sizes have shown reliable photothermal applications. BP nanomaterial exhibits an extensive spectrum of light absorption, spanning from the visible to the near-infrared (NIR) range [13,75]. This broad absorption capability enables the BP nanomaterial to effectively capture light energy from various sources commonly utilized in biomedical contexts. Consequently, BP nanomaterials possess the ability to absorb light at varying wavelengths, particularly between 550 and 590 nm (weak light absorption) and 250–450 nm (strong light absorption) [93,94]. The bandgap decreases as the number of layers increases, making few-layer BP nanomaterial suitable for visible light absorption, while monolayer BP nanomaterial is more effective for infrared light [95]. Therefore, BP nanomaterial demonstrates an impressive photothermal conversion efficiency, due to the tuneable bandgap (0.3–2.0 eV), efficiently converting absorbed light energy into heat [96,97]. This characteristic is critical for achieving rapid and precise temperature elevation within targeted tissues during photothermal therapy (PTT) and other biomedical procedures. Aksoy et al. assembled the BP nanomaterial with gold nanoparticles for photothermal antibacterial and antibiofilm activities against an important nosocomial pathogen [80]. The study reported that the antibacterial efficiency of BP nanomaterial/Au nanocomposites was significantly higher than that of the bare BP nanosheets under NIR light irradiation, suggesting a desired photoresponsivity mechanism to clear infection.

The light-to-heat conversion process, where nanomaterials can act as light absorbers and efficiently transfer light energy into heat, is termed “photothermal conversion” [98]. Materials such as BP nanomaterial can translate near-infrared light (NIR) into heat and produce a high concentration of ROS triggered by NIR irradiation. Both heating and ROS are critical to destroying the outer membranes and rupturing cell bodies, causing DNA fragmentation and leading to bacterial death [99], an important mechanism for the antimicrobial activity of BP nanomaterial. Furthermore, BP nanomaterial responsiveness to NIR light is particularly noteworthy due to its absorption peak aligning with this region of the electromagnetic spectrum [45]. The ability of NIR light to deeply penetrate biological tissues facilitates non-invasive and selective photothermal heating of target tissues, minimizing damage to surrounding healthy tissue [31,91,97]. NIR light can reach into wounds more effectively than ambient light, as the tissues scatter and absorb less light at longer wavelengths [100]. This allows for the treatment, in this case BP nanomaterial, to be applied to the wound, which then releases ROS and heat to the active site (infected wound) [101,102,103,104]. Additionally, when coupled with delivery methods such as a hydrogel, it allows for a deeper delivery of the BP nanomaterial to the active site, significantly increasing treatment efficacy [103,104].

### 4.4. The Role of BP Nanomaterial Against Bacteria

BP nanomaterial emerges as an important antimicrobial agent due to its unique properties and mechanisms of action. BP nanomaterial efficacy against a wide range of bacteria, including drug-resistant strains, is primarily attributed to the unique ability to generate ROS upon exposure to light or in an aqueous environment. These ROS, including singlet oxygen and superoxide radicals, can damage bacterial cell membranes, proteins, and DNA, leading to cell death. Additionally, the high surface area and sharp edges of BP nanomaterial contribute to its physical antibacterial activity. BP nanosheets have been demonstrated to disrupt bacterial membranes through direct contact, causing mechanical damage that compromises cell integrity, resulting in cell lysis. This dual mode of action, both chemical via ROS production and physical via membrane disruption, makes BP nanomaterial a powerful and versatile antimicrobial agent.

Preliminary studies to date have illustrated the effectiveness of BP nanomaterial against many bacterial species that remain susceptible to BP application after multiple passages. Xiong et al. 2018 showed that BP nanosheets had time- and dose-dependent bactericidal effects against Gram-negative *Escherichia coli* and Gram-positive *Bacillus subtilis* [97]. A recent study by Shaw et al. 2021 demonstrated that within two hours of microbial exposure, few-layered BP nanoflakes (BPNF) exhibited antimicrobial efficacy against a broad spectrum of bacteria and fungi, including *Pseudomonas aeruginosa*, *Cryptococcus neoformans* (sensitive, fluconazole-resistant, and amphotericin B-resistant), and methicillin-resistant *Staphylococcus aureus* (MRSA) [10]. This was further confirmed in a follow-up experiment with a eutectic gel delivery [98]. Additionally, Liu et al. 2020 reported that BP nanomaterial remained effective for over 60 days with multiple treatments against *E. coli*, whereas resistance against comparable antibiotics (which included Oxacillin, Ofloxacin, and Rifampicin) developed after only 3 days of application [8]. Similarly, Ouyang et al. (2018) found that BP nanomaterial remained effective against *S. aureus* for 10 h after treatment, while resistance to doxycycline antibiotics developed after 10 passages [9]. While bacteria can develop resistance to antibiotics and other nanoparticles (such as silver and zinc [99]), it is hypothesized that the antimicrobial mechanisms of BP nanomaterial prevent bacteria from developing resistance [27]. Additionally, trace amounts of phosphoric acid produced by BP nanomaterial oxidation may also shift bacteria out of an inactive metabolic state and help restore drug sensitivity [27]. Furthermore, BP nanomaterial oxidation produces acidic by-products that lower the wound pH, inhibiting bacterial growth and directly favoring tissue repair [11]. (2018) showed that BP nanosheets had time- and dose-dependent bactericidal effects against Gram-negative *E. coli* and Gram-positive *B. subtilis* [105]. A recent study by Shaw et al. 2021 demonstrated that within two hours of microbial exposure, few-layered BP nanoflakes (BPNF) exhibited antimicrobial efficacy against a broad spectrum of bacteria and fungi, including *P. aeruginosa*, *C. neoformans* (sensitive, fluconazole-resistant, and amphotericin B-resistant) and *MRSA* [11]. This was further confirmed in a follow-up experiment with a eutectic gel delivery [106]. Additionally, Liu et al. 2020 reported that BP nanomaterial remained effective for over 60 days with multiple treatments against *E. coli.* whereas resistance against comparable antibiotics (which included Oxacillin, Ofloxacin, and Rifampicin) developed after only 3 days of application [9]. Similarly, Ouyang et al. 2018 found that BP nanomaterial remained effective against *S. aureus* for 10 h after treatment, while resistance to doxycycline antibiotics developed after 10 passages [10]. While bacteria can develop resistance to antibiotics and other nanoparticles (such as Ag and Zn [107]), it is hypothesized that the antimicrobial mechanisms of BP nanomaterial prevent bacteria from developing resistance [28]. Additionally, trace amounts of phosphoric acid produced by BP nanomaterial oxidation may also shift bacteria out of an inactive metabolic state and help restore drug sensitivity [28]. Furthermore, BP nanomaterial oxidation produces acidic by-products that lower the wound pH, inhibiting bacterial growth and directly favoring tissue repair [12].

### 4.5. Antimicrobial Mechanism of BP Nanomaterial

**Figure 6:**
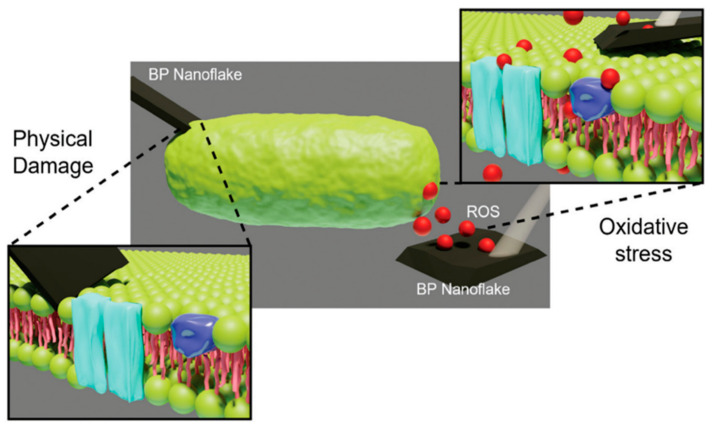
Two of the antimicrobial mechanisms of BP action: oxidative stress through ROS generation (red dots) and physical damage [32].

BP nanomaterial has two primary mechanisms of antimicrobial activity: the production of ROS and the physical properties of the nanoflakes. ROS production occurs as BP nanomaterial degrades, forming singlet oxygen (1O_2_), peroxide (O2_2_^−^), superoxide anion (O_2_^−^), hydrogen peroxide (H_2_O_2_), hydroxyl radicals (OH^•^), and hydroxyl (OH^−^) ions [11,32,50,108]. ROS are potent oxidizing agents that cause damage through oxidative stress and lipid peroxidation reactions to cellular proteins, DNA strands, RNA, and intracellular biomolecules, resulting in bacterial cell death (Figure 6) [32,50,108]. To date, studies have primarily focused on controlling the degradation of BP nanomaterial through light-activated mechanisms, specifically using blue light (405–455 nm) and UV light (100–400 nm) [20]. The activation by light sources renders this approach clinically difficult and often associated with adverse effects due to non-specific heating of the wound tissue. However, it has been demonstrated that few-layer BP nanoflakes can generate ROS in ambient light without external illumination, resulting in significant eradication of infection both in vitro and in vivo [11,12]. However, excessive ROS can also pose risks to healthy cells in the surrounding tissue if not carefully controlled, emphasizing a controlled targeted and localized approach.

The second antimicrobial mechanism of BP nanomaterial is associated with the physical properties of BP nanoflakes. The 2D structure of BP nanoflakes includes sharp edges that cause physical damage to bacterial membranes, leading to intracellular periplasmic and cytoplasmic leakage and cell death [32,53,105,108]. This process has been described as a “nanoknife” effect [53]. This “nanoknife” interaction of BP nanomaterial and pathogens has been shown in various bacteria and fungi using scanning electron microscope (SEM) and transmission electron microscope (TEM) imaging, providing evidence of pathogen membrane damage after treatment with BP nanomaterial [11,32,53,105,108]. The “nanoknife” effect is of particular importance to the antimicrobial activity of BP nanomaterial when applied in a dark environment since light is not activating the accelerated degradation of BP nanomaterial [9]. The “nanoknife” and ROS generation are evidence that BP nanomaterial has high bacterial toxicity. Unlike conventional antibiotics that target specific bacterial processes, which bacteria can adapt to over time, BP’s antimicrobial effects stem from the ability to generate reactive oxygen species (ROS) and physical damage. These mechanisms cause oxidative and physical damage to bacterial cells in a way that is broad and non-specific, attacking cell structures, membranes, and essential biomolecules simultaneously [59]. This multifaceted approach is difficult for bacteria to counteract, as multiple simultaneous adaptations would be required, unlike the single-target mechanisms of antibiotics. Moreover, BP’s potent antimicrobial activity allows for lower doses and reduced application frequency, reducing the selective pressure that often leads to resistance with prolonged antibiotic use. Hence, BP has the potential to address the challenges of antimicrobial resistance. BP nanomaterial has been demonstrated to be safe for mammalian skin cells (keratinocytes and fibroblasts) at certain concentrations, whilst the cell line and exposure time are all known to affect toxicity, as discussed in Section 3 [11,50,60]. The size difference between bacteria and mammalian cells could be a factor in the decreased toxicity towards mammalian cells; however, further research is required to better understand the underlying mechanisms of action and toxicity of BP nanomaterial [11].

The safety and compatibility of BP with human cells depends heavily on particle size and concentration. Smaller particles, with a higher surface area-to-volume ratio, degrade faster, leading to increased ROS generation. While moderate ROS levels can enhance antimicrobial effects and aid wound healing, excessive ROS from very small particles or high concentrations may harm healthy cells through oxidative stress. Similarly, controlled concentrations of BP support antimicrobial action and wound healing, but higher doses can overwhelm cellular defenses, causing cytotoxicity. Optimizing BP’s size and concentration is crucial to maximize therapeutic benefits while minimizing risks, making BP safer for clinical applications in wound management.

## 5. Emerging Role of BP in Wound Healing Applications

Wound healing is a complex biological process involving a range of cellular and molecular mechanisms aimed at restoring tissue integrity and function [109]. The properties of BP nanomaterial position it well for enhancing different stages of wound healing, including inflammation, tissue regeneration, and remodeling. One of the key properties of BP nanomaterial relevant to wound healing is its high degree of biocompatibility and minimal cytotoxicity and immunogenicity [13,31,61,110]. Studies have demonstrated that BP nanomaterial-based wound dressings promote cell proliferation and migration, which are essential processes for tissue repair and regeneration [12,13,65,77]. Moreover, BP nanomaterial biocompatibility minimizes the risk of adverse reactions, allowing for prolonged contact with the wound site without causing further damage. Additionally, acidic by-products of BP nanomaterial degradation can enhance wound healing [12,111]. This interaction with biological components creates a favorable microenvironment for tissue regeneration [12,111]. For example, Zhang and colleagues have used BP nanomaterial in microneedles for wound-healing applications [112]. The BP nanomaterial supported by an oxygen carrier has been shown to improve cutaneous healing of diabetic wounds in a diabetic rat model, suggesting a wider role in wound management [112,113]. BP nanosheets have been investigated for bio-effects on in situ skin repair. The effects of BP nanomaterial on angiogenic and anti-inflammatory abilities in vitro and in a rat wound model showed promising applications for improved skin wound healing [113].

Photothermal properties of BP nanomaterial are also important in wound management applications. By absorbing light energy, both ambient and NIR, BP nanomaterial can generate localized heat, which can promote angiogenesis and accelerate the wound healing process [13,77,114]. The controlled application of heat using BP-based materials can enhance blood flow to the wound site, facilitating nutrient and oxygen supply to the healing tissue, thereby enhancing tissue regeneration efficacy [13,77,115].

**Figure 7:**
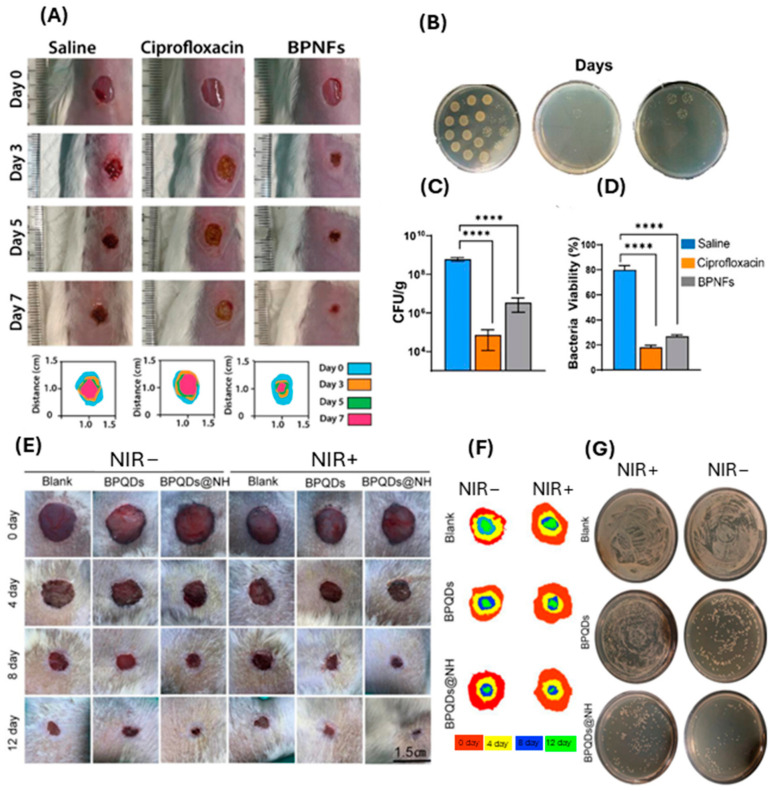
BP improves wound healing and kills bacterial species with and without NIR in in vivo models. (**A**) Representative digital photographs and schematic traced wounds demonstrating the rate of healing and wound closure. (**B**) Representative images of plates displaying colonies formed after treatment with saline, ciprofloxacin, and BPNFs respectively. (**C**) Quantification of wound bacterial levels by assessment of colony counts (CFU g^−1^) at day 7 post wounding. (**D**) Quantitative analysis of average bacteria viability from confocal images (%). * denotes significance between groups using one-way ANOVA where **** *p* < 0.0001. (Adapted from [12] with permission.) (**E**) Photographs of MRSA-infected wounds in diabetic rats and (**F**) schematic traced wounds demonstrating wound closure rates at 0, 4, 8, and 12. (**G**) CFU plates of remaining viable bacteria in tissue homogenates after treatment. (Adapted from [65] with permission.)

Chronic wounds are often prone to infections, which can significantly impede the healing process [116,117]. BP nanomaterial-based dressings have been demonstrated to possess antimicrobial activity against a wide range of pathogens, including bacteria and fungi [12,13,65,70]. By inhibiting microbial growth, BP nanomaterials create a conducive environment for wound healing and reduce the risk of infection-related complications [12,13,65,70]. The majority of in vivo studies to date have used NIR to control BP nanomaterial degradation and subsequent ROS production [28,65]. However, this was found to be detrimental to wound healing, as NIR exposure significantly increased the wound temperature (to 50 °C or above), which can result in burn injury and further delay healing [28,65]. Recently, Virgo et al. experimented on the antimicrobial and wound healing effects of few-layered BP nanoflakes in ambient light conditions (Figure 7A–D), which shows a viable alternative to NIR, an example of which is shown in Figure 7E–G [12,65].

Studies to date have also demonstrated a variety of delivery methods for BP nanomaterial applications in wound management, including microneedles [28], topical application [118], sprayed gel application [119], direct injection to the wound bed [10], and hydrogel delivery systems [65,120,121]. Current research studies are focused on optimizing delivery options of few-layered BP nanomaterial for clinical applications in the management of chronically infected wounds.

Importantly, the tuneable properties of the BP nanomaterial offer additional advantages in wound healing applications. The structure and composition of BP-based materials can be modified to achieve optimal performance for applications to specific wound types at certain stages of wound healing [17,28,45,110]. Surface functionalization techniques enable the incorporation of bioactive molecules, including growth factors and antimicrobial agents, into BP nanomaterial-based dressings, further enhancing antimicrobial properties and healing processes [17,45,110]. For BP nanomaterial to be developed as a standard wound care treatment, the stability, biocompatibility, antimicrobial effects, delivery method, and optimal concentration need to be determined and optimized. This is in addition to the manufacturing challenges associated with scalability and the logistics of mass production. Over time, and with additional research, BP nanomaterials could potentially be optimized to become the standard form of wound care management.

## 6. Future Perspectives and Challenges

The challenges of using BP nanomaterial in biomedical applications, particularly in wound healing, include scalability, reproducibility, and toxicity, all of which are critical for advancing clinical translation. Current synthesis methods often yield low amounts of BP nanomaterial with significant batch-to-batch variability, hindering large-scale production and commercialization. To address these issues, standardizing synthesis protocols, characterization techniques, and quality control measures is essential to ensure consistency and reliability. Additionally, variations in toxicity among different BP nanomaterial formulations pose a barrier to clinical application, as some formulations are highly cytotoxic. Studies have explored strategies including surface modification of BP nanomaterials, including functionalization with biocompatible polymers or encapsulation in biodegradable materials. This reduces direct cellular interactions and minimizes cytotoxic effects while enabling controlled release of therapeutics. Another strategy focuses on optimizing the oxidation rate of BP, as material toxicity is influenced by its oxidation state. By adjusting synthesis parameters or using stabilizers, researchers can enhance the stability and safety of BP nanomaterials in biological environments. Comprehensive in vitro and in vivo studies are also being conducted to establish safety profiles for different BP formulations. Through these efforts, researchers aim to mitigate the toxicity challenges associated with BP, facilitating safer and more effective clinical application of BP-based nanomaterials.

Understanding the degradation and clearance mechanisms of BP nanomaterial for both short-term and long-term applications is also critical for broad biomedical applications. Moreover, combining BP nanomaterials with other materials, including silver or zinc, to enhance antimicrobial properties introduces complexity, requiring further research, testing, and financial investment to ensure safety and efficacy. Despite these challenges, the potential of BP nanomaterial in biomedical applications remains promising. Advances in nanotechnology and materials science are expected to yield more efficient and scalable synthesis methods, improving the reproducibility and quality of BP nanomaterials. Innovations in surface modification and functionalization techniques can enhance stability and biocompatibility, reducing toxicity and broadening therapeutic applications. Ongoing research into the interaction of BP nanomaterials with biological systems will provide insights that optimize their use in clinical applications, particularly in wound management and antimicrobial therapies, ultimately enabling BP nanomaterial to reach its full potential as a versatile agent in biomedicine.

## 7. Conclusions

BP nanomaterial is showing promise for diverse biomedical applications, particularly in wound healing. This review provides a comprehensive overview of the synthesis, characteristics, and biomedical applications of BP nanomaterial, with a specific focus on its potential role in promoting wound repair and tissue regeneration. By elucidating the fundamental properties of BP nanomaterial, including its two-dimensional structure, interactions with biological systems, and therapeutic mechanisms, there are unique opportunities for BP nanomaterial in biomedical applications.

The future of BP nanomaterial in regenerative medicine is promising, but as mentioned above, several challenges and opportunities lie ahead. Continued interdisciplinary research collaborations will be essential for advancing the development and translation of BP nanomaterial-based therapies, enabling the design of multifunctional materials tailored for specific biomedical applications. Tackling the scalability and reproducibility challenges in BP synthesis and manufacturing is essential for achieving large-scale production and commercialization of BP nanomaterial-based biomedical products. Overcoming these obstacles will demand advancements in nanotechnology and materials science, as well as the establishment of standardized protocols and regulatory frameworks to ensure consistent quality and safety. Moreover, exploring the combination of BP nanomaterials with other therapeutic agents and materials can further enhance BP efficacy and broaden the range of biomedical applications. This includes the development of advanced drug delivery systems, antimicrobial treatments, and imaging technologies that leverage the unique properties of BP nanomaterial.

In conclusion, BP nanomaterial holds promise as a versatile platform for biomedical applications, with the potential to revolutionize wound healing strategies and advance regenerative medicine. Despite the many challenges and the need for additional research, BP nanomaterial possesses unique properties and capabilities that can provide innovative solutions to unmet needs in wound care and introduce a new era of personalized and precision medicine. Continued dedication to overcoming these obstacles will ultimately unlock the full potential of BP nanomaterial, leading to significant advancements in healthcare.

## Figures and Tables

**Table 1:** Examples of studies where the cytotoxicity of BP has been investigated.

Type of BP Used	Concentration	Cell Type Tested Against	Key Observation	Reference
BP nanosheets	25–200 µg/mL	4T1, HeLa, L929, A549	Minimal cytotoxicity	[67]
BP nanoparticles with dextran (DEX) and branched poly(ethyleneimine) (PEI), and folic acid (FA)	3.125–100 µg/mL	4T1, 3T3	Minimal cytotoxicity	[68]
BP nanosheets with polydopamine (PDA), and zinc oxide (ZnO) nanowires on titanium (Ti) substrates	20 mg/mL	MC3T3-E1, L929	Minimal cytotoxicity	[58]
BP nanosheets with ε-poly-l-lysine (ε-PL)	0.8–50 µg/mL	293T, HaCaTs	Minimal cytotoxicity	[69]
BP nanosheets	12.5–200 µg/mL	HeLa, HepG-2, MCF-7, L02	Minimal cytotoxicity	[53]
BP quantum dots in hydrogel (NH)	50–500 µg/mL	HUVECs, HaCaTs	Minimal cytotoxicity	[65]
BP nanosheets with silk fibroin (SF)	50–500 µg/mL	HSF	Minimal cytotoxicity	[70]
BP sheets	3.125–400 µg/mL	A549	Intermediate cytotoxicity	[62]
BP nanodots	0.05–3.0 mg/mL	HeLa, COS-7, CHO-K1	Minimal cytotoxicity	[25]
BP nanoflakes	312.5–5000 µg/mL	HaCaTs, HFF	Minimal–Intermediate cytotoxicity	[12]
Layered BP	6.25–200 µg/mL	NIH 3T3, HCoEpiC, 293T	Minimal–High cytotoxicity	[63]
BP nanosheets	0.3–125 µg/mL	NIH3T3, nHDF, HT1080	Minimal–High cytotoxicity	[60]
